# Adalimumab plus Conventional Therapy versus Conventional Therapy in Refractory Non-Infectious Scleritis

**DOI:** 10.3390/jcm11226686

**Published:** 2022-11-11

**Authors:** Binyao Chen, Shizhao Yang, Lei Zhu, Xuening Peng, Daquan He, Tianyu Tao, Wenru Su

**Affiliations:** 1 State Key Laboratory of Ophthalmology, Zhongshan Ophthalmic Center, Sun Yat-sen University, Guangzhou 510060, China; 2 Guangdong Provincial Clinical Research Center for Ocular Diseases, Guangzhou 510060, China

**Keywords:** adalimumab, refractory scleritis, glucocorticoids, immunosuppressants, TNF-α inhibitor

## Abstract

Long-term systemic glucocorticoids and non-specific immunosuppressants remain the mainstay of treatment for refractory scleritis, and result in serious side-effects and repeated inflammation flares. To assess the efficacy and safety of additional adalimumab, patients diagnosed with refractory non-infectious scleritis were enrolled. They were assigned to the conventional-therapy (CT, using systemic glucocorticoids and other immunosuppressants) group or the adalimumab-plus-conventional-therapy (ACT) group according to the treatments they received. The primary outcome was time to achieve sustained remission, assessed by a reduction in modified McCluskey’s scleritis scores. Other outcomes included changes in McCluskey’s scores, scleritis flares, best-corrected visual acuity, and spared glucocorticoid dosage. Patients in the ACT group achieved faster remission than those in the CT group, as the median periods before remission were 4 months vs. 2.5 months (*p* = 0.016). Scleritis flares occurred in 11/11 eyes in the CT group and 5/12 eyes in the ACT group (*p* = 0.005). Successful glucocorticoid sparing was realized in both groups, but the ACT group made it faster. No severe adverse events were observed. Data suggest that adalimumab plus conventional therapy could shorten the time to remission, reduce disease flares, and accelerate glucocorticoid withdrawal compared with conventional therapy alone.

## 1. Introduction

Non-infectious scleritis is thought to be an immune-mediated inflammation of the outer layer of the eyeball, referred to as sclera [1]. Its clinical manifestations range from insidious ocular discomfort and redness to insufferable and radiating pain, and irreversible visual loss [2,3,4]. Briefly, non-infectious scleritis can be divided into anterior scleritis or posterior scleritis, and the former can be further subdivided into diffuse, nodular, and necrotizing scleritis, based on the anatomic site and clinical appearance of inflammation [3]. As reported previously, scleritis typically affects middle-aged individuals and considerably more frequently affects women [2,5]. Systemic autoimmune diseases, including rheumatoid arthritis (RA), ANCA-associated vasculitis, and relapsing polychondritis, are common in patients with scleritis [4,6]. Authentic data on the prevalence of scleritis are difficult to obtain, varying from 0.08% to 8.7% depending on the reporting institutions [4,7]. Although rare, the recurrent and prolonged course, along with multiple ocular complications, including keratitis, uveitis, scleral staphyloma, corneal necrosis, and scleral perforation, might compromise visual function and even lead to blindness [8,9].

Evidence indicates that aberrant immune system and inflammatory response play crucial roles in the pathogenesis of scleritis [10,11]. Until recently, non-specific immunosuppressants were the mainstay of non-infectious scleritis treatment [2]. Topical and oral glucocorticoids (GCs), along with other immunosuppressants including methotrexate (MTX), mycophenolate mofetil (MMF), and cyclosporine A (CsA), are commonly used to treat severe cases of scleritis. However, in refractory cases, limited clinical success and intolerant side effects caused by the long-term medication underscore the need for new targeted biological agents [12,13]. 

Tumor necrosis factor (TNF)-α is an inflammatory mediator that plays multiple pathogenic roles in scleritis by expanding the inflammatory response, stimulating the production of autoantibodies, and promoting the release of tissue-destructive matrix metalloproteinases [10]. Researchers have found that TNF-α levels are elevated in the serum and aqueous humor of patients with ocular inflammatory diseases [14]. Adalimumab, a fully human monoclonal anti-TNF-α antibody, has achieved satisfactory clinical success in treating non-infectious uveitis and systemic autoimmune diseases [15,16]. It has been approved for the treatment of multiple systemic inflammatory diseases, including RA, juvenile idiopathic arthritis, ankylosing spondylitis, Crohn’s disease, ulcerative colitis [17,18,19], and severe ocular inflammatory conditions such as intermediate, posterior, and pan-uveitis [20]. Regarding scleritis, several case reports and uncontrolled case series have made tentative explorations of the effectiveness of the off-label use of adalimumab [21,22]. A controlled clinical trial is warranted to clarify the therapeutic effect and safety of adalimumab combined with conventional therapy in the treatment of refractory scleritis. This retrospective study aimed to systemically compare the efficacy and safety of conventional therapy (CT group, using GCs and other immunosuppressants) and adalimumab plus CT in patients with refractory non-infectious scleritis.

## 2. Materials and Methods

### 2.1. Patient Population

This retrospective study was performed in accordance with the guidelines of the Declaration of Helsinki. Approval was granted by the Ethics Committee of Zhongshan Ophthalmic Center (2020KYPJ104), and informed consent was obtained from all patients. Medical records of patients diagnosed with non-infectious scleritis at Zhongshan Ophthalmic Center from August 2020 to August 2022 were reviewed. 

Patients were included if they: (1) were diagnosed with chronic non-infectious scleritis (including anterior and posterior scleritis). Anterior scleritis was diagnosed based on the characteristic clinical symptoms of painful inflammation of the sclera and periocular tenderness that radiated to the forehead, physical signs of scleral edema and congestion of the deeper episcleral vessels, and pathomorphological changes in ultrasound biomicroscopy. The episcleral and scleral tissues are both edematous and congestive, and congestion of the deep scleral vessels remains after the application of phenylephrine drops; patients with episcleritis were excluded from this study [4]. Posterior scleritis was diagnosed based on clinical symptoms of periocular pain, blurred vision, conjunctival chemosis, and B-mode ultrasonographic changes, including increased scleral thickness, T sign, scleral nodules, and other abnormal signs [23]; (2) still suffered from recurrent ocular pain and redness and even progressive visual loss when attending the clinic, despite the fact that therapeutic doses of oral GC and/or immunosuppressive medication had been administered for at least 4 weeks, which required an increased dose of oral GC or additional immunosuppressive drugs to control the episode; (3) had received at least 6 months of regular treatment and attended the clinic every four weeks during follow-up. 

Patients were excluded if they (1) had received prior treatment with anti-TNF-α therapies or other biologic agents; (2) had any evidence of neoplasia or systemic infections (e.g., human immunodeficiency virus, hepatitis, or active tuberculosis); (3) were pregnant; (4) had a history of poorly controlled medical conditions (e.g., uncontrolled diabetes mellitus, renal disease), or poor general health.

### 2.2. Treatment Protocols

All patients underwent a systemic examination, including complete blood count, comprehensive metabolic panel, chest computed tomography, hepatitis B and C, human immunodeficiency virus serology, T-SPOT, and a purified protein derivative (PPD) skin test to exclude the contraindications of systemic immunosuppressive therapy. Additional testing, including rheumatoid factor, antinuclear antibody, human leukocyte antigen-B27 (HLA-B27), and antineutrophil cytoplasmic antibody, was performed in certain patients when positive signs or symptoms were found in the examination or review of systems to search for underlying systemic diseases. 

Patients diagnosed with latent tuberculosis, defined as T-SPOT+ or PPD+ without radiographic or clinical evidence of disseminated or pulmonary disease, received preventative anti-tuberculosis treatment to reduce the risk of tuberculosis reactivation associated with TNF-α inhibition or oral GCs. 

The risks, benefits, and alternatives of adalimumab were explained to all the patients. Based on medical records, patients were assigned to the conventional-therapy (CT) group or the adalimumab-plus-conventional-therapy (ACT) group according to the treatment they received, using traditional immunosuppressive medication (including oral GCs and other immunosuppressants) or CT plus regular adalimumab injections. 

Patients enrolled had been diagnosed with scleritis and received standardized systematic immunosuppressive treatments (GCs and other immunosuppressants) for at least 4 weeks. The onset time of this study was defined as the date they visited the clinic for the first time. After the examination, the regular therapy went into effect. 

A course of high-dose oral GCs (prednisone) was administered at enrollment when patients were in the active disease period. The starting daily dose of prednisone ranged from 0.5 to 1.0 mg per kilogram of body weight, combined with immunosuppressive agents, usually CsA (50–150 mg/day), MTX (7.5–15 mg/week), or MMF (1–3 g/day). Under assessment, the dosage of GCs could be tapered once the disease was controlled. In general, oral GCs were tapered ahead of concomitant immunosuppressive agents and were maintained at the lowest dosage that controlled scleritis activity. 

Adalimumab was supplied in prefilled syringes and administered subcutaneously. After a baseline loading dose of 80 mg, adalimumab was administered every two weeks at a dosage of 40 mg. The regular adalimumab injection lasted for no less than six months. 

Severe disease flares were treated with an escalated dose of oral GCs, and limited flares were treated with an increase in the doses of MTX, CsA, or both. 

### 2.3. Follow-Up

The follow-up records included patients’ reported symptoms and clinical presentations as inspected by qualified ophthalmologists. Patients were required to attend the clinic every four weeks or whenever they felt unwell. The evaluation at the first visit, when patients received the initial dose of adalimumab or reset high dose of GCs, was used as baseline for all analyses.

Regular evaluations included systemic and ocular history, best-corrected visual acuity (BCVA) on a standard logarithmic visual acuity chart, slit-lamp examination, intraocular pressure measurement, dilated fundus examination, and laboratory tests. Furthermore, the number and dosage of systemic and regional GCs and immunosuppressive agents were recorded. B-ultrasound examinations were performed at regular intervals on patients diagnosed with posterior scleritis. Patients were asked to report any ocular or systemic discomfort during treatment. The clinicians were masked for the purpose of conducting the study at the time of inclusion and follow-up.

### 2.4. Clinical Endpoints

Disease activity was measured using a modified McCluskey’s scleritis disease grading scale [24], based on specified parameters of ocular signs and symptoms, including (1) the number of inflamed scleral quadrants (scores from 0 to 4), (2) degree of globe tenderness (graded from 0 to 4), (3) presence or absence of nodules, (4) presence or absence of corneal involvement, (5) degree of anterior chamber cells, (6) degree of vitreous cells, (7) presence or absence of retinal detachment, (8) presence or absence of optic nerve swelling, and (9) presence or absence of increased scleral thickness or T-sign inB ultrasound (Table A2). Scores ranged from 0 to 23 points, with higher scores indicating more severe scleritis activity. The modified McCluskey’s scores for each eye were evaluated by two qualified ophthalmologists based on the medical records. 

The primary efficacy endpoint was the time to achieve sustained disease remission, defined as a reduction in McCluskey’s scores by at least 4 points or to 0 for at least 2 months [25]. 

Secondary outcome measures included McCluskey’s scores at the last visit, changes in McCluskey’s scores from baseline to the last visit, proportion of eyes with flares, time to first flare, rates of flares per eye, rates of flares per eye per month, changes in BCVA, and spared GC dosage. Flares were defined as an increase in McCluskey’s scores by at least two points. The time to achieve sustained remission and the time to first flare were calculated using the Kaplan–Meier method. The BCVA tested on standard logarithmic visual acuity chart was converted to logMAR (logarithm of the minimal angle of resolution), and, to be measurable and comparable, the very low visions, scaled as “finger counting (FC)/hand motion (HM),” were quantitated as 1.85/2.30 LogMAR [26]. 

Adverse events (AEs) were monitored during the study period. Adalimumab injections were stopped once severe AEs were reported. It is suggested to stop adalimumab in patients with sustained remission for at least 24 months.

### 2.5. Statistical Analysis 

The time to achieve sustained remission and the time to first flare were calculated using the Kaplan–Meier method. Descriptive statistics are presented as mean ± standard deviation (SD), median (interquartile range, IQR), or numbers (percentages). Regarding the comparisons of baseline demographics, clinical characteristics, and outcome measures between the two groups, normality of the distribution was assessed using the Shapiro–Wilk test, and quantitative data were compared using Student’s *t*-test or Mann–Whitney U test, as appropriate. Fisher’s exact test was performed for categorical variables. SPSS software (version 26; IBM Corp., Armonk, NY, USA) was used for statistical analysis. Statistical significance was set at *p* < 0.05.

## 3. Results

### 3.1. Baseline Characteristics

Eighteen patients with non-infectious, non-necrotizing scleritis met the inclusion criteria and were retrospectively analyzed in this study, with nine patients in each group. No patient was censored due to severe AEs or sustained remission. As certain patients were diagnosed with bilateral scleritis, a total of 23 eyes were included (11 eyes in the CT group and 12 eyes in the ACT group). All patients were Han Chinese. Detailed demographic characteristics and disease-related information at baseline are summarized in Table 1. The data are presented by individuals in Table A1. Baseline characteristics were similar between the two groups. The ocular diagnoses included anterior scleritis (eight patients in the CT group and five in the ACT group), posterior scleritis (zero in the CT group and two patients in the ACT group), and panscleritis (one in the CT group and two in the ACT group). Panscleritis refers to a third type of scleritis, whereby inflammation occurs in both the anterior and posterior segments, as proposed by Wieringa et al. [9]. The sex ratios (male/female, 3/9 in the CT group and 4/9 in the ACT group) and mean age at the onset of scleritis (38.44 years in the CT group and 31.22 years in the ACT group) were not significantly different between the two groups. On average, the duration from disease onset to recruitment was 13.56 months in the CT group and 7.89 months in the ACT group was (*p* = 0.142). A minority of the patients were diagnosed with binocular scleritis (3/9 in the CT group and 2/9 in the ACT group). All patients were followed up for longer than six months, with an average period of 13 months in the CT group and 12.11 months in the ACT group. At baseline, as the patients were in the active period of acute exacerbation, a high dosage of oral GCs was administered, with an average of 30 mg/day in the CT group and 31.65 mg/day in the ACT group. Concomitant immunomodulators administered at baseline, including MTX, CsA, and MMF, were also documented. In terms of systemic immune-related diseases, one patient in the ACT group was diagnosed with connective tissue disease and two patients in the CT group were diagnosed with erythema nodosum and leukoderma. 

### 3.2. Outcome Measures

The outcomes of McCluskey’s scores and BCVA were measured by eye, while the GC dosage assessment was documented by persons (Table 2).

**Figure 1:**
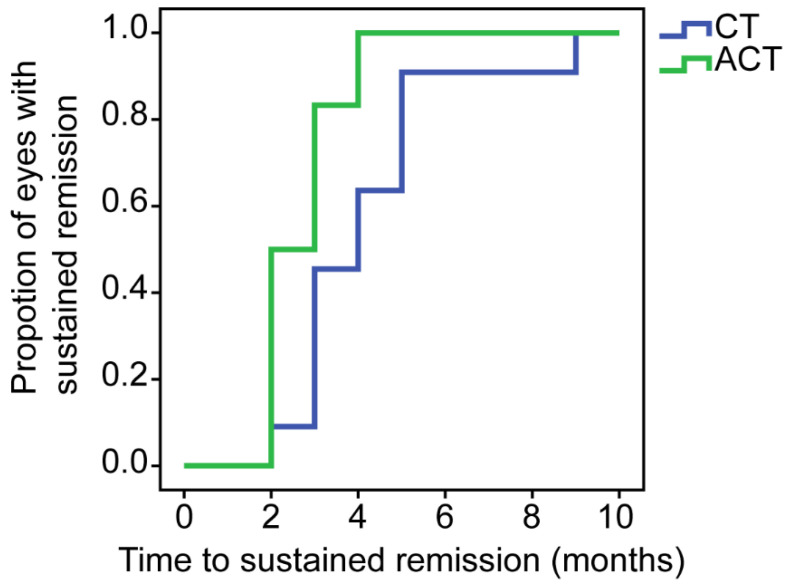
Kaplan–Meier curve of time to sustained remission of scleritis since baseline. In the 4th month, 100% of the ACT (adalimumab plus conventional therapy) group achieved sustained remission, and in the 9th month, 100% of the CT (conventional therapy) group achieved that.

At baseline, all the eyes had active scleritis. The baseline McCluskey’s scores were not significantly different between the two groups (8 ± 2.569 in the CT group vs. 8.42 ± 2.392 in the ACT group, *p* = 0.691), while the McCluskey’s scores at last visits showed a significant difference between the two groups (*p* = 0.032). The decrease in McCluskey’s scores from baseline to the last visit was 4.364 ± 2.618 in the CT group and 7.25 ± 2.734 in the ACT group. The changes in McCluskey’s scores were larger in the ACT group (*p* = 0.017). During the treatment, all eyes in the two groups achieved sustained remission (decrease in McCluskey’s scores ≥4, or overall McCluskey’s scores = 0 for at least two months). The patients in the ACT group achieved faster remission than those in the CT group, as the median period before sustained remission was 4 months vs. 2.5 months (*p* = 0.016), respectively. The time to achieve sustained remission is also shown by the Kaplan–Meier curve in Figure 1. 

**Figure 2:**
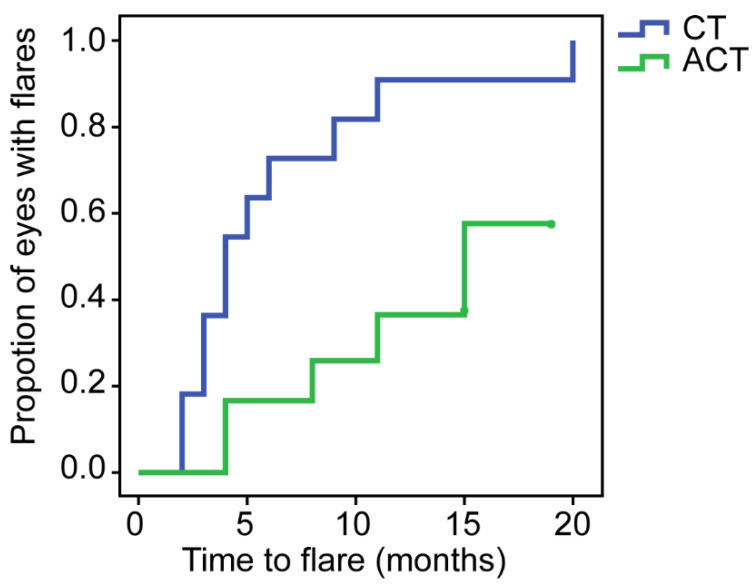
Kaplan–Meier curve of time to flare of scleritis since baseline. In the 20th month, flares occurred in 100% of the eyes in the CT (conventional therapy) group, and 7/12 (58.33%) eyes in the ACT (adalimumab plus conventional therapy) group remained without flares.

Scleritis flares (defined as an increase in McCluskey’s scores ≥2) occurred in 11/11 eyes in the CT group and 5/12 eyes in the ACT group during follow-up (*p* = 0.005). The median time to first flare was 4 months (in the CT group) and 11.5 months (in the ACT group) from baseline (*p* = 0.019). The Kaplan–Meier curve shows a longer duration of no-flare-survival in the ACT group (Figure 2). Notably, 7/12 eyes in the ACT group had no flare throughout the follow-up period. Two eyes in the CT group experienced flares more than once. After calculation, the median number of flares per eye per month was 0.125 in the CT group and 0 in the ACT group. The median number of relapses per eye in the CT group was 1 (1, 1), and that in the ACT group was significantly lower (0, [0, 1], *p* = 0.006).

**Figure 3:**
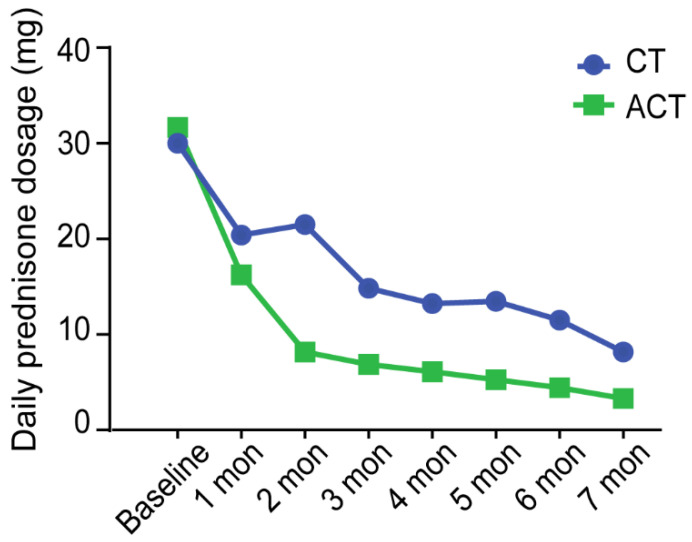
Variation in daily oral prednisone dosage per patient in the ACT (adalimumab plus conventional therapy) group and in the CT (conventional therapy) group during treatment.

The starting dosages of oral GCs were similar in the two groups (30 ± 5.449 mg in the CT group vs. 31.67 ± 24.206 mg in the ACT group, *p* = 0.845), as the patients were all in the active period at baseline. The dosage was adjusted according to the disease activity during the following visits. Changes in GC dosage during follow-up are presented in Figure 3. Until the seventh month, the average GC dosage was reduced to less than 10 mg/day in both groups, which realized successful GC sparing as defined by the Standardization of Uveitis Nomenclature (SUN) Working Group [27]. However, the ACT group achieved a faster reduction in GC dosage.

**Figure 4:**
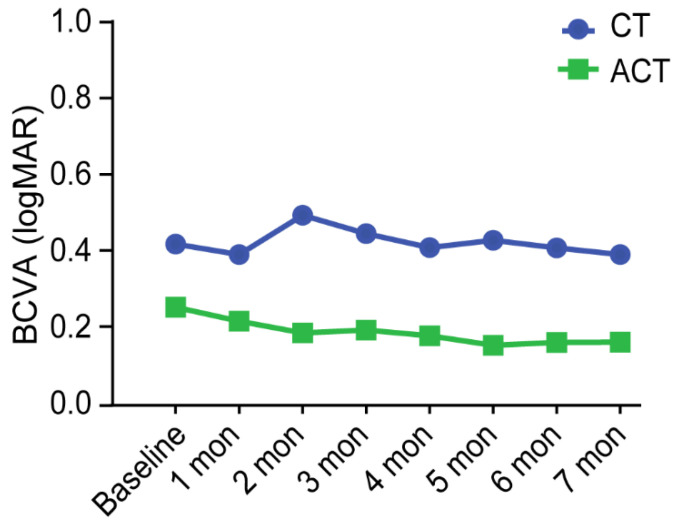
Variation in BCVA (LogMAR) per eye in the ACT (adalimumab plus conventional therapy) group and in the CT (conventional therapy) group during treatment.

Although baseline visual acuity was higher in the ACT group than in the CT group, the difference was not statistically significant (0.097 vs. 0.201, *p* = 0.134). There was little change in visual acuity during treatment in both groups (Figure 4).

In total, three AEs were reported in the ACT group during the follow-up period, among which no safety issues necessitated either a temporary hold or early termination of adalimumab treatment (Table 3). None of the patients in either group developed opportunistic infections. No severe AEs, including malignancies, congestive heart failure, or multiple sclerosis/neurological diseases, were reported. One patient in the ACT group had injection site reactions characterized by mild erythema, pain, and swelling at the injection site, which was bearable and did not necessitate discontinuation of adalimumab. Another patient in the ACT group reported erythema multiforme-like skin reactions on the face, and two upper arms developed erythema after the seventh injection of adalimumab. Joint pain was also documented in one patient in the ACT group.

## 4. Discussion

Refractory scleritis is a rare but sight-threatening immune-mediated ocular inflammation that might seriously impair visual function and quality of life with recurrent attacks [9,28]. The mainstream first-line agents for scleritis, including systemic GCs and immunosuppressants, sometimes fail to achieve lasting remission of inflammation, which contributes to a dose-dependent risk of adverse effects from long-term GC use [2]. Adalimumab, a novel TNF-α inhibitor, has been proven to be effective in long-term remission of ocular inflammation, sparing systemic GCs, and rescue of visual acuity, mostly by experience and studies on uveitis [29,30,31].

Herein, we report on the efficacy and safety of adalimumab plus CT for the treatment of refractory non-infectious scleritis. The results underscore the following three points: (1) adalimumab plus conventional immunosuppressants shortens the time to achieve remission, prolongs the period of sustained remission, and reduces disease flares effectively compared with CT; (2) adalimumab accelerates GC withdrawal during controlled inflammation; (3) AEs of adalimumab are endurable in most patients with refractory scleritis in the short-term. 

Successful management of disease progression as soon as possible and suppression of flares are crucial for the prognosis and quality of life. TNF-α inhibitors have the advantage of rapid onset of inflammation control. In patients with an acute attack of panuveitis, a single infliximab infusion reduced intraocular inflammation faster than either intravitreal triamcinolone acetonide or intravenous methylprednisolone, with maintenance therapy with oral GCs and immunosuppressants [32]. In a randomized placebo-controlled trial in patients with steroid-dependent inactive uveitis, adalimumab significantly lowered the risk of uveitic flares during the withdrawal of GCs [33]. In the present study, 100% of patients in the CT group experienced recurrence during follow-up, the earliest of which occurred in the second month. Comparatively, favorable control of flares was observed in the ACT group (flares occurred in 41.67% of patients). One patient with binocular scleritis remained relapse-free for 19 months following regular adalimumab injections. Moreover, adalimumab was found to accelerate GC tapering and maintain inflammation being inactive. Long-term systemic GC treatment, which is commonly required in patients with refractory scleritis, is often complicated by various AEs such as renal insufficiency, hypertension, leukopenia, thrombocytopenia, and hepatic toxicity [34]. In this regard, additional use of adalimumab could speed up the control of inflammation and taper oral GCs, thus avoiding irreversible ocular complications and side effects. In addition, although it is conventional to combine immunosuppressants and adalimumab in most studies, some evidence has suggested that combination therapy added no benefit in ocular inflammation control and glucocorticoid-sparing [35,36]. Further evidence is needed to confirm the effectiveness and safety of monotherapy of ADA without immunosuppressants in scleritis patients.

The key role of TNF-α in the pathogenesis of inflammatory diseases has been emphasized in previous studies, which provides the basis for anti-TNF-α agents for treating scleritis [10,13]. Although a complete understanding of immunopathogenesis is challenging, non-infectious scleritis is generally accepted as an immune complex-mediated immune disorder, or a local delayed hypersensitivity reaction [37], in which both innate and adaptive immunity might be involved. As a powerful inflammatory cytokine, TNF-α plays an important role in the onset, duration, and expansion of scleral inflammation [11]. It can be synthesized by and affects various cell types such as macrophages, monocytes, T-helper cells, plasma cells, neutrophils, and endothelial cells [38]. Subsequently, TNF-α can activate the production of matrix metalloproteinases from infiltrating inflammatory cells and stromal scleral fibroblasts, leading to scleral necrosis [39,40]. Previous research has found increased TNF-α levels in the tear fluid and scleral tissue of patients with active scleritis [40,41,42], but not in the blood [43]. A recent analysis of a mouse model of arthritis-associated scleritis revealed that macrophages, plasma cells, and deposition of immune complexes jointly participate in the pathogenesis of scleritis [44], and suggested targeting molecular targets, such as TNF-α and IL-6, inhibiting macrophage activity, and CD20, suppressing antibody-producing cells, instead of targeting T-cells. In addition, a study on the genotypes of TNF-α-related genes in Chinese Han patients with scleritis found that specific haplotypes in TNFAIP3 (TNF-α-induced protein 3), TNFSF4 (TNF-αreceptor superfamily member 4), and TNFSF15 might be the risk or protective factors of scleritis in Chinese Han [45]. In summary, the evidence supporting the critical role of TNF-α in scleritis provides a basis and rationale for TNF-α-targeting treatment.

Therefore, TNF-α inhibitors have been used to treat ocular inflammatory diseases. Adalimumab, a fully humanized monoclinal anti-TNF-α antibody, has been approved for the treatment of non-infectious intermediate, posterior, and panuveitis in adults and pediatric patients 2 years of age and older by the Food and Drug Administration. Based on a systemic review of published evidence, it is recommended as a first-line immunomodulatory agent for ocular manifestations of Behçet’s disease, a first-line nonsteroidal agent for uveitis associated with juvenile arthritis, and potential first-line non-steroidal agent for severe posterior uveitis, panveitis, and uveitis associated with seronegative spondyloarthropathy [46]. A three-center retrospective case series, including 60 patients with uveitis, reported a positive effect of adalimumab in 82% of patients, independently of additional systemic disease and uveitis type [47]. In patients with Behçet’s vasculitis, both naïve and refractory, adalimumab plus CT outperformed CT alone, regarding the symptomatic improvements [48,49]. As for Vogt–Koyanagi–Harada, a retrospective study showed that additional adalimumab was effective and safe in patients refractory to CT alone [50]. Even adalimumab plus immunosuppressants could be considered as a systemic GC-free therapy in treatment-naïve Vogt–Koyanagi–Harada patients [51]. For scleritis, there are several case reports and case series of non-infectious scleritis successfully treated with adalimumab [21,22,52,53]. Lawuyi et al. [21] reported two cases of refractory necrotizing scleritis, remaining quiet on two-weekly adalimumab treatment for over six months. In a patient of nodular scleritis with RA, intolerant to systemic steroids, complete resolution of ocular inflammation was achieved [22]. Adalimumab was also reported to realize rapid control of scleritis within 3 months with no recurrence over 5 years in a patient with recurrent idiopathic bilateral nodular scleritis [52]. Several studies have evaluated the curative effect of TNF-α inhibitors, including infliximab and adalimumab, in patients with ocular inflammation, including uveitis and scleritis, without subgroup analysis [54,55,56,57]. A retrospective review of 17 patients with refractory non-necrotizing scleritis received infliximab or/and adalimumab treatment, found 88% patients achieved inactive inflammation for at least 2 months [58]. According to available evidence, adalimumab treatment has a promising effect on ocular inflammation control or GC sparing regardless of systemic autoimmune disease association. However, there is no trial-based evidence to support these data, except for the experience provided by case reports and case series. To our knowledge, this is the first comparative study to assess the effects of adalimumab in refractory scleritis. This study has reinforced previous evidence for the effectiveness of adalimumab in controlling ocular inflammation and GC sparing.

Currently, clinical studies on the state of scleritis are inconsistent and nonstandard, and some of them subjectively documented the severity as “active/inactive”, thus sharply reducing the credibility and comparability between groups of patients treated by different protocols. A standard point-scoring system that systemically evaluates inflammatory activity and severity is needed to optimize the assessment of therapeutic effects and standardize the clinical course of scleritis. In 1991, McCluskey et al. [24] proposed a quantitative scoring system, covering the common clinical signs of scleritis, for grading the severity of scleritis and monitoring the response to therapeutic effect in all types of scleritis except for scleromalacia perforans. McCluskey et al. described each item at length to guide the research to a proper score, and defined improvement as a decrease in the score by four or more points. Few studies on treatment of scleritis adopted this grading system [25,59]. Sen et al. [60] also designed a digital photograph-based scleritis grading system, mainly based on vascular changes in the sclera. However, few studies have adopted this system [61]. This study adopted McCluskey’s system and modified the parameters by adding the main clinical signs of posterior scleritis, including increased scleral thickness or the T-sign in B-ultrasound and optic nerve swelling [23]. The clinical symptoms of posterior scleritis are insidious, often leading to severe damage and loss of vision. This additional point assignment highlighted the importance of posterior scleritis [62]. The multicomponent primary endpoint by modified McCluskey’s system assesses various facets of scleritis, covering patients’ subjective feelings, clinical signs and symptoms, and objective test results. However, the modified scoring system requires validations from larger cohort studies. The reliability and applicability need to be verified in future studies of scleritis. The safety profile of adalimumab in the present study was similar to that of other approved indications [63]. The main reported AE are injection site reactions, which are commonly encountered. More serious potential AEs, such as infection, including reactivation of latent tuberculosis and cytopenias, might be avoided by systemic medical examination of patients prior to treatment initiation. Rare side effects, including worsening of congestive cardiac failure, increased risk of malignancy, and exacerbation or development of demyelinating disease, were not observed in this study, due to the limited cases and short period. No new safety signals were observed.

The limitations of this study include the retrospective and open-label design as well as the limited number of included patients and follow-up period. A potential selection bias exists due to the retrospective design. Moreover, the inclusion of patients with bilateral scleritis could bring in a bilaterality bias, as the analysis assumed that observed events were independent. Randomized controlled trials are required to adequately assess the long-term clinical efficacy and safety profile of adalimumab for refractory scleritis. It is declared that, as the enrolled population is all Chinese Han, the relative homogeneity of the cohort does not allow to generalize our findings in other ethnic groups.

## 5. Conclusions

In conclusion, obtained findings suggest that adalimumab plus CT could be a well-tolerated and effective treatment option for patients with refractory non-infectious scleritis, to accelerate inflammation control, reduce scleritis flares, and promote GC tapering.

## Figures and Tables

**Table 1:** Patients’ demographics and clinical characteristics.

Characteristic	CT	ACT	*p*-Value
Number of patients	9	9	
Number of eyes	11	12	
Male, n (%)	3 (33.3%)	4 (44.4%)	1
Age at onset, mean ± SD (years)	38.44 ± 10.806	31.22 ± 15.328	0.265
Interval before baseline, mean ± SD (months)	13.56 ± 9.774	7.89 ± 4.512	0.142
Site of scleritis, n (%)			0.149
Anterior scleritis	8	5	
Posterior scleritis	0	2	
Panscleritis	1	2	
Binocular, n (%)	2(22.2%)	3 (33.3%)	1
Sytemic immune-related diseases, n (%)	2 (22.2%)	1 (11.1%)	1
Follow-up duration, mean ± SD (months)	13 ± 5.937	12.11 ± 3.655	0.708
Baseline oral GC dosage, mean ± SD (mg/day)	30 ± 5.449	31.67 ± 24.206	0.845
Baseline concomitant immunomodulators			0.71
MTX	6	8	
CsA	5	8	
MMF	3	2	

CT, group of conventional therapy; ACT, group of adalimumab plus conventional therapy; SD, standard deviation; GC, glucocorticoid; MTX, methotrexate; CsA, cyclosporine A; MMF, mycophenolate mofetil.

**Table 2:** The parameters of outcomes.

Outcome Measures	CT	ACT	*p*-Value
Baseline McCluskey’s scores, mean ± SD	8 ± 2.569	8.42 ± 2.392	0.691
McCluskey’s scores at the last visit, median (IQR)	2 (2,6)	0 (0, 1.75)	0.032
Changes in McCluskey’s scores, mean ± SD	−4.364 ± 2.618	−7.25 ± 2.734	0.017
logMAR BCVA, median (IQR)	0.201 (0.097, 0.398)	0.097 (0, 0.374)	0.26
logMAR BCVA at the last visit, median (IQR)	0.201 (0.097, 0.699)	0.048 (0, 0.301)	0.134
Changes in logMAR BCVA, median (IQR)	0 (−0.201, 0.104)	−0.049 (−0.097, 0)	0.651
Eyes with sustained remission, n (%)	11 (100%)	12 (100%)	
Time to achieve sustained remission, median (IQR), months	4 (3, 5)	2.5 (2, 3)	0.016
Eyes with flares, n (%)	11 (100%)	5 (41.67%)	0.005
Time to first flare, median (IQR), months	4 (3, 9)	11.5 (7.25, 14.75)	0.019
Flare times per eye per month, median (IQR)	0.125 (0.091, 0.143)	0 (0, 0.083)	0
Flare times per eye, median (IQR)	1 (1, 1)	0 (0, 1)	0.006

CT, group of conventional therapy; ACT, group of adalimumab plus conventional therapy; logMAR, logarithm of the minimal angle of resolution; BCVA, best-corrected visual acuity; SD, standard deviation; IQR, interquartile range.

**Table 3:** The adverse events in the 2 groups.

Documented Adverse Events	CT	ACT
Any adverse event	0	3
Adverse event leading to death	0	0
Adverse event leading to discontinuation of study drug	0	0
Injection-site reactions	0	1
Malignancies	0	0
Opportunistic infections	0	0
Demyelinating disease	0	0
Lupus-like reaction	0	0
Erythema multiforme-like skin reactions	0	1
Joint pain	0	1

CT, group of conventional therapy; ACT, group of adalimumab plus conventional therapy.

## Data Availability

Not applicable.

## Appendix A

**Table A1:** Demographics and clinical characteristics of individual patients.

Group	No.	Age at Onset	Sex	OD/OS/OU	Interval before Baseline, m	Site of Scleritis	Systemic Immune- Related Diseases	Follow-Up, m	Dates of Onset
CT	1	46	F	OD	22	anterior		21	Aug, 2020
	2	47	F	OD	24	anterior	erythema nodosum	9	Dec, 2020
	3	58	M	OS	10	anterior		8	Mar, 2021
	4	39	M	OD	1	anterior	leukoderma	22	Aug, 2020
	5	31	F	OU	24	anterior		7	Jul, 2020
	6	41	M	OD	8	anterior		15	Jul, 2020
	7	32	F	OD	5	panscleritis		17	Sep, 2020
	8	28	F	OS	4	anterior		11	Sep, 2020
	9	24	F	OU	24	anterior		7	Mar, 2021
ACT	10	49	M	OD	2	anterior		12	Jan, 2021
	11	15	M	OU	15	posterior		14	Aug, 2020
	12	39	F	OD	10	anterior		15	Jun,2020
	13	40	F	OU	9	anterior		12	Jun, 2020
	14	19	M	OU	10	posterior		19	Jan, 2020
	15	56	F	OS	2	anterior		12	Oct, 2020
	16	28	F	OS	3	anterior	connective tissue disease	7	May, 2021
	17	15	M	OS	10	panscleritis		10	Mar, 2021
	18	20	F	OD	10	panscleritis		8	Jan, 2021

CT, group of conventional therapy; ACT, group of adalimumab plus conventional therapy; OD, Oculus Dexter; OS, Oculus Sinister; OU, Oculus Uterque.

**Table A2:** The modified McCluskey’s scleritis disease grading scale.

Clinical Features	Score Range	Score Definition
Tenderness *	0–4	0–4
Nodules	0–1	0 for absent, 1 for present
Corneal involvement @	0/2/4	0 for absent, 2 for present, 4 for progressive
Anterior chamber cells	0/1/2	0 for no cells, 1 for ≤20 cells/field, 2 for >20 cells/field
Vitreous cells	0/1/2	0 for no vitreous haze, 1 for grade 1/2 haze, 2 for grade 3/4 haze
retinal detachment	0/2	0 for absent, 2 for present
increased scleral thickness or T sign in B-mode ultrasound	0/2	0 for absent, 2 for present
optic nerve swelling	0/2	0 for absent, 2 for present

* Adapted from Peter McCluskey et al. [24]. @ Includes acute stromal keratitis, sclerosing keratitis, peripheral corneal melts, and marginal corneal ulcers.
